# Cell body clustering drives gap junction-mediated synchronous activity in command neurons

**DOI:** 10.64898/2026.02.26.708359

**Published:** 2026-03-02

**Authors:** Kristen Lee, Josmarie Graciani, Natalie Rico Carvajal, Zhehao Zhu, Matt Q Clark, Chris Q Doe

**Affiliations:** 1 Institute of Neuroscience, Howard Hughes Medical Institute, University of Oregon, Eugene, OR 97403; 2 Biology Department, Bucknell University, Lewisburg, PA 17837

## Abstract

The nervous system contains densely packed cell bodies, yet the role of neuronal cell body position in circuit function is poorly understood. Here we show that four *Drosophila* Moonwalker Descending Neurons (MDNs), command neurons for backward locomotion, must maintain cell body contact to allow gap junction-dependent synchronous activity necessary to initiate backward walking. MDNs express the transcription factor Hunchback, which drives expression of the Lar cell adhesion molecule; Hunchback, Lar, and its ligand Dlp promote MDN cell body clustering and backward walking. When clustered, the gap junction protein Inx8 allows synchronous firing of MDNs, which is required to initiate backward walking. These findings reveal a previously unappreciated role for cell body clustering and synchronous firing in neural circuit function.

Neuronal electrical activity must be precisely regulated to avoid excitotoxicity or failure to activate circuits (1). Proper neuronal communication allows the brain to integrate environmental cues and generate motor output. Neuronal circuits are often depicted as one neuron communicating with another, although neurons are rarely functioning alone. Typically, multiple neurons make up a neuronal type, which communicates with other neuronal types (i.e. other neuronal ensembles) (2). However, little is known about the principal features needed for neurons comprising a neuronal type to function properly within a neuronal network. Neuronal features such as morphology, neurotransmitter choice, and ion channel composition all have the potential to modify neuronal activity and connectivity (3). Here we add one more attribute to this list: cell body positioning to regulate electrical coupling.

To identify features regulating circuit activity, we used the well-characterized *Drosophila* Moonwalker Descending Neuron (MDN) escape circuit, which commands backward walking (4). There are four MDNs, two on each side of the midline, which respond to environmental stimuli before synapsing with neurons in the brain and ventral nerve cord (VNC) to initiate and maintain backward walking (5–10). The third thoracic segment of the ventral nerve cord primarily controls the hindlegs. Within this neuropil, MDNs major output is onto the pre-motor neurons LBL40 and LUI130 that drive backward walking (8).

The MDN circuit is uniquely equipped to investigate the relationship between neuronal activity and neuronal identity within a multi-neuron ensemble. We find a single transcription factor, Hunchback (Hb), is required in the four MDNs for expression of the Lar-Dlp cell adhesion complex, which generates a tight four-cell body cluster of MDNs. Strikingly, without cell body clustering, MDN-induced backward walking does not occur. This system allows us to address a novel question within the field of neuroscience: what role does cell body location have for neuronal circuit function? We find that cell body clustering of MDNs is necessary for synchronous firing of all four MDNs via gap junction electrical synapses, thereby identifying a previously undescribed feature of neuronal types necessary for network activity. It is possible that cell body location may be an important principal for neuronal network function across species; electrical synapses are well-documented to be present within populations of a single neuron type, including the fly lamina, worm AVA interneurons, and mammalian inferior olive neurons (11–13). Here, our data support a model where synchronous activity, enabled by clustered cell bodies, is required to translate diverse inputs into a temporally coherent burst of activity that is required for initiating backward walking.

## Hunchback is required in MDNs for backward walking

**Figure 1.**
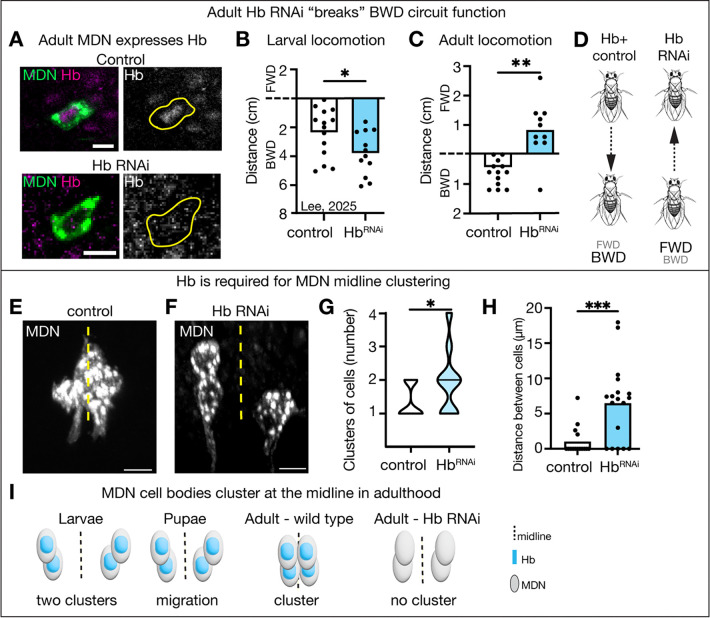
Hunchback is required for adult MDN behavior and cell body clustering at the midline. **(A)** Hb expression is lost when the Hb RNAi transgene is expressed in MDN. Scale bar, 5 μm. **(B)** Data from Lee, 2025(14); distance traveled when MDN is activated in larvae. **(C)** Distance traveled when MDN is optogenetically activated in adults. **(D)** Summary of behavior results. **(E-F)** Adult MDN cell body position relative to midline (dashed). Scale bar, 5 μm. **(G-H)** Quantification of E-F. **(I)** Cartoon representation of results. Statistical analyses were performed using unpaired two-sided t-test (*p<0.05, **p<0.01, ***p<0.001, ****p<0.0001).

We previously showed that MDNs express Hb across larval life (14). Here, we show that MDNs maintain Hb expression into adulthood (Fig. 1A). As anticipated, expressing *UAS-Hb*^*RNAi*^ produces a strong Hb knockdown in adult MDN neurons (Fig. 1A) (15). Hb knockdown in larval MDNs produced an increase in MDN-induced backward locomotion (Fig. 1B), due to an increase in synaptic connectivity with A18b, a downstream partner in the backward circuit (14). To determine the function of Hb in the adult MDN, we knocked down Hb in post-mitotic MDNs and assayed walking behavior. The adult MDN driver turns on during mid-metamorphosis.

In control animals, when adult MDNs are optogenetically activated, the animals walk backwards (Fig. 1C) (4, 16). Unexpectedly, when Hb is knocked down in adult MDNs, optogenetic activation failed to elicit backward locomotion (Fig. 1C), thereby “breaking” the MDN circuit (Fig. 1D). Thus, Hb is regulating some adult MDN feature that is required to induce backward walking.

One possible explanation for the locomotor difference between larvae and adults would be neurotransmitter switching. The larval MDNs are excitatory cholingeric neurons (4, 17), and Hb knockdown maintains ChAT expression and lacks GABA inhibitory neurotransmitter expression in adult MDNs (fig. S1A, B), indicating that Hb is regulating a different MDN feature required for backward walking. To determine if the MDNs have synapse abnormalities, we assayed MDN chemical synapse number. Loss of Hb did not alter MDN synapse number (fig. S1C-E). To determine if the MDNs have defective morphology, we assayed the morphology of single-labeled MDN neurons via Multi-Colored Flip Out (18). Loss of Hb did not alter MDN axon or dendrite morphology (fig. S2).

In larvae, two pair of MDNs are located off the midline; during metamorphosis the cell bodies move towards the midline before forming a tight four-cell cluster in adults (Fig. 1I; fig. S1C). Interestingly, Hb knockdown impaired clustering of MDN cell bodies at the midline (Fig. 1E-H). In contrast, Hb knockdown had no effect on MDN cell body position in the larvae (fig. S1F-H). We conclude that aggregation of MDN cell bodies at the midline during adulthood is disrupted in Hb knockdown (Fig. 1I).

## Hunchback promotes Lar expression, which is required for MDN midline clustering and backward locomotion

**Figure 2.**
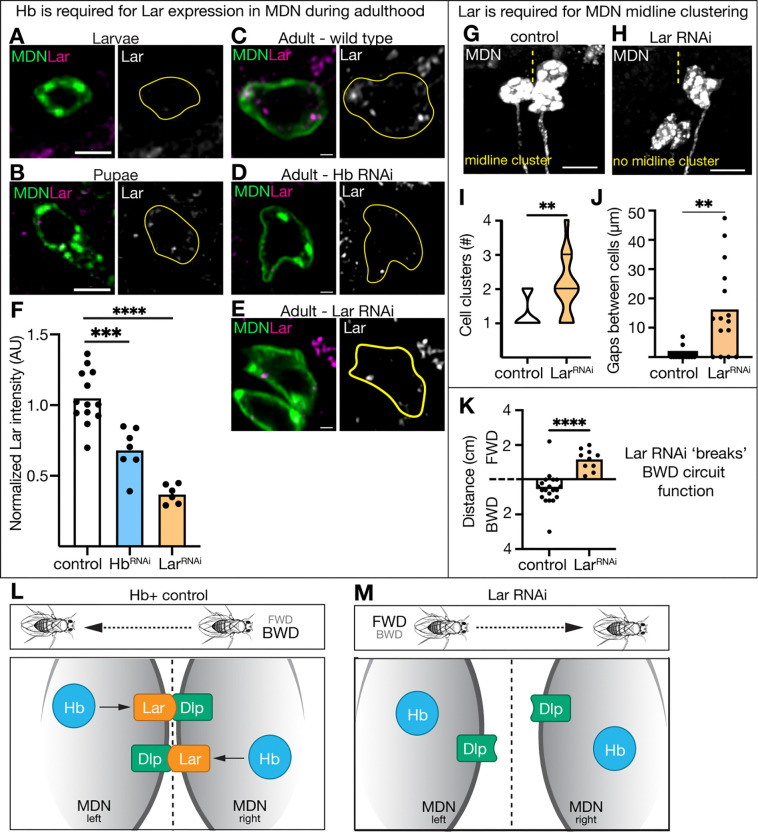
Hunchback promotes Lar expression, which is required for MDN midline clustering and backward locomotion. (A-C) Lar expression in MDN at indicated stages. A and B: scale bar, 5 μm. C: scale bar, 1 μm. **(D)** Lar expression is reduced when Hb RNAi is expressed in MDN. Scale bar, 1 μm. **(E)** Lar expression is reduced when Lar RNAi is expressed in MDN. Scale bar, 1 μm. **(F)** Quantification of C-E. **(G-H)** Adult MDN cell body morphology. Scale bar, 7 μm. Yellow dash line represents the midline. **(I, J)** Quantification of G-H. **(K)** distance traveled when MDN is optogenetically activated. **(L, M)** Cartoon representation of results. Statistical analyses were performed using a two-way ANOVA with Bonferroni’s multiple comparisons or an unpaired two-sided t-test, as appropriate (*p<0.05, **p<0.01, ***p<0.001, ****p<0.0001).

To determine whether loss of Hb or lack of MDN cell body clustering resulted in loss of MDN-induced backward walking, we screened for guidance cues that prevented midline clustering without altering Hb levels. We found that the Type IIa receptor-like protein tyrosine phosphatase Lar was expressed in MDNs during pupation and adulthood, but not in larvae (Fig. 2A-C). Next, we asked whether Lar was important for MDN cell body clustering. Expression of *UAS-Lar*^*RNAi*^ in the adult MDN neurons resulted in a strong Lar knockdown (Fig. 2E, F) and disrupted midline clustering of MDNs (Fig. 2G-J) without altering Hb levels (t-test, p=0.842). We then asked if Hb was required for the expression of Lar; adult Hb knockdown in MDN resulted in significantly reduced Lar (Fig. 2D, F). We then assayed Lar knockdown to see if disruption of the MDN midline clustering, but not loss of Hb levels, resulted in failure to walk backwards upon optogenetic activation. Control animals walked backward when MDN was optogenetically activated, whereas Lar knockdown animals failed to go backwards (Fig. 2K). We conclude that Lar knockdown in MDNs “breaks” the backward locomotion circuit, without altering Hb levels.

Lar has well-characterized roles in neuronal circuit wiring (19). Our data suggest a novel role of Lar: attracting neuronal cell bodies to the midline. To further demonstrate that Lar is required for MDN cell bodies to cluster at the midline, we assayed Lar ligands: N-cadherin (CadN), Sticks and stones (Sns), Syndecan (Sdc), and Dally-like protein (Dlp) (19–22). Like Lar, Dlp was expressed in pupal and adult MDNs but not in larvae (fig. S3A-C). Additionally, we investigated whether Dlp was important for MDN cell body clustering. We observed a moderate Dlp knockdown when expressing *UAS-Dlp*^*RNAi*^ in the adult MDN neuron (fig. S3E, F), yet the partial Dlp knockdown still prevented MDN midline clustering without altering Hb levels (fig. S2G-J; t-test, p=0.691). Conversely, knocking down Hb in adult MDNs did not alter Dlp expression (fig. S3D, F). Taken together, these data lead to a linear model: Hunchback promotes Lar expression, Lar promotes MDN midline clustering by binding to Dlp, and MDN clustering is required for MDN-induced backward locomotion (Fig. 2L, M).

## Innexin 8 is required for MDN clustering and initiation of backward walking

The requirement of MDN cell body clustering for backward walking was surprising. How might disrupting the MDN soma cluster lead to failed backward walking induction? One possibility is that all four MDNs are gap junction coupled, resulting in adhesion between the cell bodies and synchronous firing. It is well-documented that electrical synapses can occur between cell bodies, regulate adhesion, and allow neurons to fire synchronously (23, 24).

**Figure 3.**
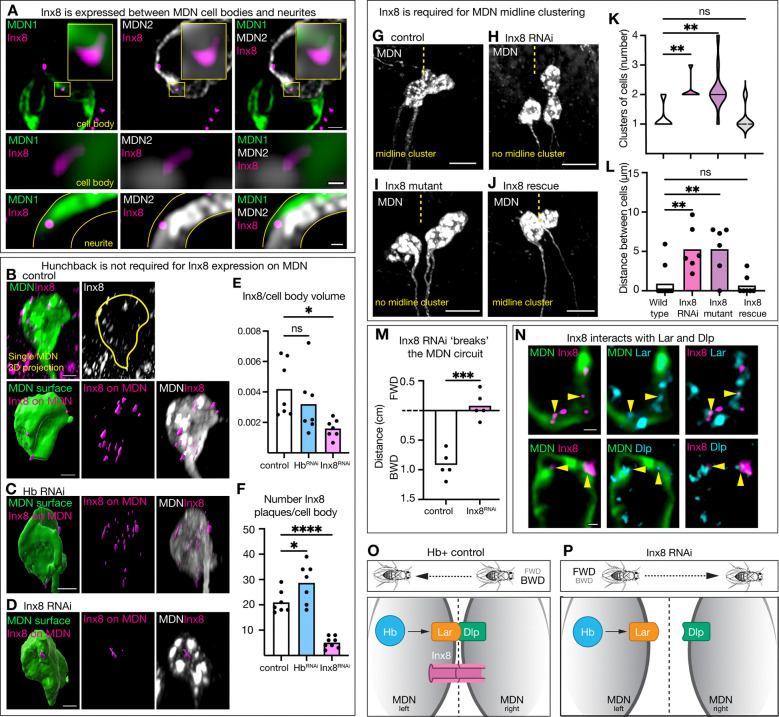
Innexin 8 is required for MDN midline clustering. **(A)** Inx8 is present between individual MDN cell bodies and neurites. Top: scale bar, 1 μm. Middle: scale bar, 0.1 μm. Bottom: scale bar, 0.3 μm. **(B-D)** Inx8 puncta expression on MDN cell bodies when Hb RNAi and Inx8 RNAi is expressed in MDN. Scale bar, 3 μm. **(E, F)** Quantification of B-D. **(G-J)** Adult MDN cell body morphology. Scale bar, 10 μm. Yellow dash line, midline. **(K, L)** Quantification of G-J. **(M)** Distance traveled when MDN is optogenetically activated. **(N)** Inx8 expression alongside Lar (top) and Dlp (bottom) on MDN cell bodies. Scale bar, 0.5 μm. **(O, P)** Cartoon representation of results. Statistical analyses were performed using a two-way ANOVA with Bonferroni’s multiple comparisons or an unpaired two-sided t-test, as appropriate (*p<0.05, **p<0.01, ***p<0.001, ****p<0.0001).

We assayed the widely expressed gap junction component Innexin 8 (Inx8; Flybase: ShakB) (25). We confirmed that Inx8 is detected in adult MDNs, specifically between MDN cell bodies and between primary neurites (Fig. 3A), with little at axon terminals (fig. S4A). We then tested whether Hb was required for Inx8 expression. We found that Hb knockdown did not remove Inx8 expression in MDNs but led to an increased number of smaller Inx8 puncta compared to control (Fig. 3B-F; see Discussion). We observed a similar result when Lar was knocked down (fig. S4B-E). Next, we assayed the role of Inx8 in MDNs. We expressed *UAS-Inx8*^*RNAi*^ specifically in the MDNs, as well as assaying a homozygous viable *Inx8* null mutant. In both experiments we found significantly reduced Inx8 expression on MDNs (Fig. 3D-F), suggesting that MDN-MDN gap junctions are reduced or eliminated in these genotypes, which inhibited MDN cell body clustering (Fig. 3G-L). Importantly, MDN-specific expression of a wild-type Inx8 transgene in the *Inx8* mutant background fully rescued MDN midline clustering (Fig. 3J-L). Lastly, we investigated whether Inx8 knockdown disrupts backward walking upon MDN optogenetic activation. In this experiment, control animals went backward when MDNs were activated, whereas Inx8 knockdown animals paused (Fig. 3M). Given that Inx8, Lar, and Dlp are all cell surface molecules, we investigated whether they are associated on MDNs cell membrane. We found that Inx8 puncta were associated with both Lar and Dlp (Fig. 3N). We conclude that Inx8, Lar, and Dlp are interacting on MDN cell bodies to promote MDN clustering, which is required for proper behavioral output (Fig. 3O, P). We conclude that Innexin 8 is required for MDN clustering and initiation of backward walking.

## MDN clustering is required for synchronous firing

**Figure 4.**
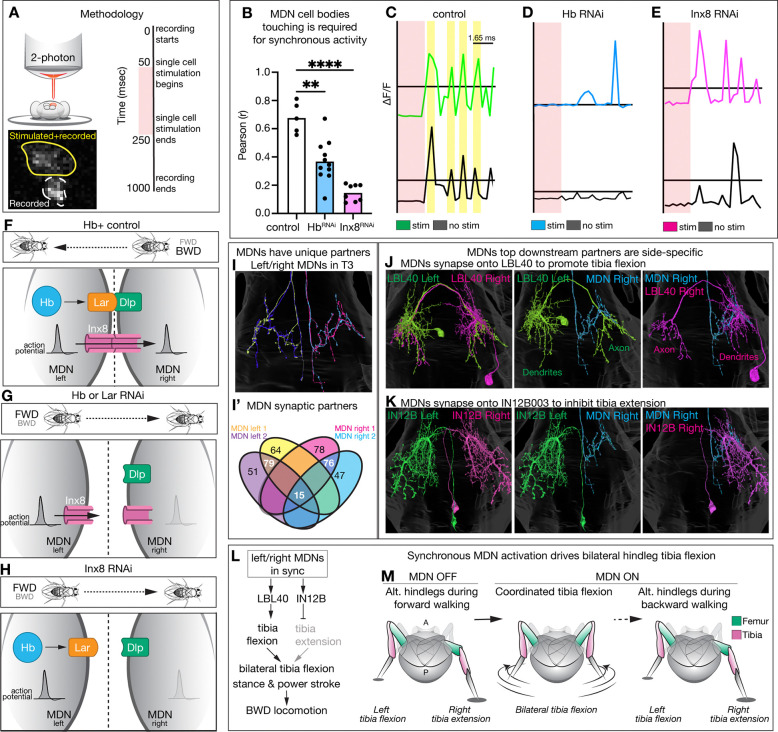
MDN clustering is required for synchronous firing, which drives initiation of backward walking. **(A)** Methodology for the genetically encoded voltage indicator experiments. **(B)** Pearson correlation coefficients of the likelihood adjacent MDN will fire when the stimulated MDN fires. Statistical analyses were performed using a two-way ANOVA with Bonferroni’s multiple comparisons (*p<0.05, **p<0.01, ***p<0.001, ****p<0.0001). **(C-E)** Voltage indicator traces between stimulated cell (colored) and adjacent cell (black) in a single animal when Hb or Inx8 RNAi is expressed in MDN. Yellow bands demarcate synchronous activity. Black horizontal lines represent resting membrane potential. **(F-H)** Cartoon representation of results. **(I)** Each individual MDN, and its downstream partners, is represented by a unique color. **(J)** Right MDN only forms synapses with the right LBL40, not the left. **(K)** Right MDN only forms synapses with the right IN12B003, not the left. **(L)** Neuronal circuit downstream of all four MDNs firing synchronously. **(M)** initiation of backward walking requires coordinated bilateral hindleg flexion.

We hypothesized that synchronous activity of the four MDNs is required for MDN-induced backward locomotion. We tested for synchronous firing in control, Hb knockdown, and Inx8 knockdown animals using the genetically encoded voltage indicator ASAP5 (26). We expressed the red-light gated cation channel, CsChrimson, in MDNs, and used two-photon microscopy to activate a single MDN. We then measured the voltage response of both the stimulated MDN and an adjacent non-stimulated MDN (typically 2 total cells; Fig. 4A). For each animal we calculated the likelihood of the non-stimulated MDN to fire synchronously with the stimulated MDN; we found that control MDNs were significantly more likely to have a synchronous voltage response compared to Hb and Inx8 knockdown animals (Fig. 4B). Overall, control non-stimulated cells frequently fired synchronously with the stimulated cell (Fig. 4C), while the non-stimulated Hb and Inx8 knockdown MDNs had much smaller voltage fluctuations and infrequent synchronous activity (Fig. 4D-E; population quantification in fig. S4F-H). Additionally, Hb knockdown in MDNs had a lower resting membrane potential, likely due to increased electrical coupling with neighboring inhibitory GABAergic cells (27) (fig. S4I-K). Taken together, we conclude that MDN clustering is required for synchronous firing and regulating membrane potential, which is required for MDN-induced backward walking (Fig. 4F-H).

## Synchronous MDN activity is required for initiation of backward walking

How does failure of MDN to fire synchronously result in a loss of backward locomotion? In wild type, initiation of backward locomotion is triggered by a brief, bilateral hind leg flexion, followed by a bilateral backward power stroke, before alternating leg movements characteristic of backward walking (8, 10). We hypothesized that loss of synchronous MDN activation might generate an uncoordinated hindleg flexion, preventing backward walking initiation. This model predicts that the four MDNs have a diverse population of output neurons that require synchronous MDN firing to generate robust, bilateral hind leg movement. Consistent with this hypothesis, we identified MDN downstream partners using the Male Adult Nerve Cord connectome (28), and found that the four MDNs had very few common outputs (3.6% or 15 total), with hundreds of outputs targeted by individual MDNs or left/right pairs (Fig. 4I, I’). The neurons with the most synapses were the premotor neurons LBL40 and IN12B003 (Fig. 4J, K), which are unique to MDN left/right pairs (Fig. 4I’). LBL40 activation triggers hindleg flexion, whereas IN12B003 inhibits hindleg extension (8, 10). Even these neurons have diverse outputs: LBL40 targets the contralateral neuropil, while IN12B003 remains on the ipsilateral side, highlighting how synchronous activation of the left/right MDNs is needed to activate bilateral tibia flexion while suppressing bilateral tibia extension (Fig. 4L, M). In our experiments, knockdown of Hb, Lar, or Inx8 abolishes MDN synchronous firing and fails to initiate backward walking. We propose that loss of MDN synchronous activation results in the uncoordinated activity of the diverse population of MDN output neurons, preventing coordinated bilateral tibia flexion and failure to initiate backward locomotion.

## Discussion

Our findings highlight the importance of cell body position for neuronal circuit function. We find that anatomical location of cell bodies and expression of ligand-receptor cell adhesion proteins, along with gap junction electrical synapses, are required for MDNs to initiate backward walking. Notably, these functions are independent of MDN chemical synapses and axon/dendrite morphology. The transcription factor Hb activates expression of the ligand-receptor pair, Lar-Dlp, which is required for the MDNs to form a single cell body cluster at the midline (Fig. 1, 2). The four MDN cell bodies form electrical synapses with each other, allowing them to fire synchronously (Fig. 3, 4). Disrupting cell body location by knocking down Hb, Lar, or Inx8 inhibits MDN-induced backward locomotion (Fig. 1–3). Moreover, we show that each MDN forms downstream connections with a mostly non-overlapping pool of neurons. Thus, MDN synchronous activation is required to coordinately activate each of the “microcircuits” downstream of MDN, which are essential for backward walking (Fig. 4)(8).

Our observation that Hb promotes neuronal cell body adhesion to allow synchronous firing is a novel result. Hb is a well-characterized transcription factor that generates early-born neuronal identity in *Drosophila* (29) and mammalian cortex and retina (30, 31). In the *Drosophila* larvae, Hb is required for post-mitotic MDN morphology and chemical synapse number (14). Here we show that during adulthood, Hb plays no role in MDN axon/dendrite morphology (fig. S2) or chemical synapse number (fig. S1C-E).

We find that Hb actives expression of the cell adhesion molecule Lar in pupal and adult MDNs (Fig. 2). During pupal stages, the four MDN cell bodies require Lar-Dlp to cluster together. Outside of the CNS, Lar plays a role in cell migration in the follicular epithelium (32, 33). Additionally, we may have uncovered a new function of Lar in supporting electrical synapse plaque size. Proteomic work in Zebrafish shows that cell adhesion molecules are in close proximity to Mauthner electrical synapses (34), but the relationship between electrical synapses and cell adhesion molecules has not been tested *in vivo*. Here we find that MDN electrical synapses are in close proximity with the cell adhesion molecules Lar and Dlp, and we show that reducing Lar expression (via Hb or Lar knockdown) leads to smaller electrical synapse puncta on MDN (Fig. 3; fig. S4), a novel result within the gap junction field. These data support the hypothesis that the transmembrane protein Lar is required for Inx8 organization at MDN-MDN contact sites. The likelihood that cell adhesion molecules are required for gap junction organization or stability is provocative; to date, most *in vivo* experiments have only characterized adherens junctions and scaffold protein interactions with gap junctions (24, 35). Given that fly innexin gap junctions can function in vertebrate cells, and vertebrate connexin gap junctions can function in invertebrates (36, 37), these results are likely translatable to other systems.

Although gap junctions are found in non-neuronal cells (38, 39), electrical synapses are present in escape circuits across species. In *C. elegans*, electrical synapses are present between the left and right AVA interneurons, which command forward and backward locomotion (40). In this system, electrical coupling is required for asymmetric inhibitory and excitatory sensory input to be processed and integrated into the correct motor response (13, 40) – a striking example of the importance of synchronous activity between a neuron ensemble. The *Drosophila* Giant Fiber neurons control a jumping escape response and forms electrical synapses with downstream motor neurons that are required for escape behavior (41, 42). Interestingly, the transmembrane protein Frazzled is necessary for both Giant Fiber morphogenesis and gap junction development (43). The Mauthner cell in Zebrafish initiates a fast C-bend escape behavior and has pre- and post-electrical synapses with upstream interneurons (44). The left/right Mauthner cells are strategically not synchronous in this neural network; unilateral firing allows precise directional turning away from aversive stimuli (45). Like the Giant Fiber and Mauthner cell circuits, most previous studies assaying the influence of gap junctions on behavior focus on electrical coupling between distinct cell types (46–49). These examples highlight the importance of neuronal ensembles being electrically coupled. Gap junctions are not formed promiscuously between random adjacent cells (50, 51) and cAMP has been shown to be a crucial regulator of electrical synapse specificity (52). Our data not only add to this growing body of literature but demonstrate the absolute requirement of proper electrical coupling within a neural network for behavior: without synchronous activity of the four MDNs, the fly is incapable of initiating backward walking.

Previous work used a genetic technique to activate left or right MDNs, finding that asymmetric activation still initiated backward walking but skewed the fly to turn to the contralateral side (5). We believe that their results complement our study; here, we find that each individual MDN forms synapses with diverse populations of downstream neurons. Therefore, due to the electrical coupling between MDNs, there is a mechanism defining how asymmetric activation of MDN could elicit backward walking. Sen et al (5) theorized “each of the four MDNs can act independently and that collectively they control both the magnitude and direction of backward locomotion”. Our data demonstrate that MDNs do not work independently. The majority of the shared neurons downstream of MDN are side-specific (Fig. 4), and Cheong et al (10) proposed that side-specific inhibition of a front leg interneuron downstream of MDN likely contributes to turning. We agree that all four MDNs collectively control the initiation of backward walking. Here, even though all four MDNs are exposed to light for optogenetic activation at the same time, cell body clustering is required for normal circuit output. We believe a combination of factors contribute to this - such as resting membrane potential differences, gap junction regulation of response magnitude, and coordinated downstream circuit activation – demonstrating the requirement of coupling between the four MDNs (fig. S5). Taken together, our study has implications not just in the understanding of *Drosophila* motor output, but also in recognizing cell body location as a novel regulator of neural output.

## Supplementary Material

Supplement 1

Materials and Methods

Figs. S1 to S5

## Data Availability

All data will be deposited in databases for open access, and/or by request.
