# Supplementary material for: Cell body clustering drives gap junction-mediated synchronous activity in command neurons
Source: bioRxiv. 2026 Mar 2:2026.02.26.708359. Preprint. [Version 1] doi: 10.64898/2026.02.26.708359 (PMC13001486; doi:10.64898/2026.02.26.708359)
Supplement: Supplement 1 [file NIHPP2026.02.26.708359v1-supplement-1.pdf]

bioRxiv preprint doi: https://doi.org/10.64898/2026.02.26.708359; this version posted March 2, 2026. The copyright holder for this preprint (which was not certified by peer review) is the author/funder, who has granted bioRxiv a license to display the preprint in perpetuity. It is made available under aCC-BY 4.0 International license.

Supplementary Materials for

Cell body clustering drives gap junction-mediated synchronous activity in command neurons

Authors: Kristen Lee[1*], Josmarie Graciani[1], Natalie Rico Carvajal[1], Zhehao Zhu[2], Matt Q Clark[2], Chris Q Doe[1*]

*Corresponding authors: cdoe@uoregon.edu, klee4@uoregon.edu

This PDF file includes:

Materials and Methods
Figs. S1 to S5
References

Materials and Methods

Fly husbandry: All flies were reared in a 25C room at 50% relative humidity with a 12 hour light/dark cycle. All flies used in experiments were 4-5 day old adults reared on standard cornmeal food medium. All comparisons between groups were based on studies with flies grown, handled, and tested together.

Fly strains: Short name, source, and full genotype of each individual fly strain used in this study is outlined below. Detailed genotypes for each experiment are provided in the corresponding methods section.

MDN-Gal4 (Bidaye, 2014[1]): VT50600.p65AD (attp40)/CyO; VT44845.Gal4DBD(attp2)/TM3,Ser or SS03131-Gal4
Control RNAi (BDSC# 31603): ;; UAS-Luc RNAi TRiP.JF01355 (attp2)
Hb RNAi (BDSC# 34704): ;; UAS-Hb RNAi TRiP.HMS01183 (attp2)
Lar RNAi (BDSC# 40938): ; UAS-Lar RNAi TRiP.HMS02186 (attp40);
Dlp RNAi (BDSC# 50540): ; UAS-Dlp RNAi TRiP.GLC01658 (attp40);
Inx8 RNAi (BDSC# 27292): ;; UAS-shakB RNAi TRiP.JF02604 (attp2)
Inx8 mutant (Desplan Lab): ShakB2;;
Inx8 rescue (Desplan Lab): ShakB2; UAS-ShakB; UAS-ShakB
Chrimson (BDSC# 55134): UAS-CsChrimson.mVenus (attp18);;
MCFO (BDSC# 55134 and 77140): hs-FlpG5::Pest; 10X UAS(frt.Stop)myr::smGDP-V5-THS-10XUAS(frt.Stop)myr::GDP-Flag;
ASAP5 (Clandinin Lab and BDSC# 55134): UAS-Chrimson::mVenus (attp8); UAS-ASAP5/CyO; TM2/TM6
LBL40-LexA (Feng, 2020[2]): ; VT021418-LexAGADPI (attp40);
Reporters used (BDSC# 64092): LexAop-myr::smGdp-V5, UAS-myr::smGdp-HA;;

bioRxiv preprint doi: https://doi.org/10.64898/2026.02.26.708359; this version posted March 2, 2026. The copyright holder for this preprint (which was not certified by peer review) is the author/funder, who has granted bioRxiv a license to display the preprint in perpetuity. It is made available under aCC-BY 4.0 International license.

Reporters used (BDSC# 32197): ;;UAS-myr::GFP

Hb, Lar, Dlp, and Inx8 knockdown experiments: The following genotypes were utilized for these experiments.
Control knockdown genotype: LexAop-myr::smGdp-V5, UAS-myr::smGdp-HA; VT50600.p65AD; VT44845.Gal4DBD/ UAS-Luc RNAi TRiP.JF01355.
Hunchback knockdown genotype: LexAop-myr::smGdp-V5, UAS-myr::smGdp-HA; VT50600.p65AD; VT44845.Gal4DBD/ UAS-Hb RNAi TRiP.HMS01183
Lar knockdown genotype: LexAop-myr::smGdp-V5, UAS-myr::smGdp-HA; VT50600.p65AD/UAS-Lar RNAi TRiP.HMS02186; VT44845.Gal4DBD
Dlp knockdown genotype: LexAop-myr::smGdp-V5, UAS-myr::smGdp-HA; VT50600.p65AD/UAS-Dlp RNAi TRiP.GLC01658; VT44845.Gal4DBD
Inx8 knockdown genotype: LexAop-myr::smGdp-V5, UAS-myr::smGdp-HA; VT50600.p65AD; VT44845.Gal4DBD/ UAS-shakB RNAi TRiP.JF02604

Innexin 8 mutant and rescue experiments: The following genotypes were utilized for these experiments.
Innexin 8 mutant: ShakB2; VT50600.p65AD; VT44845.Gal4DBD/UAS-myr::GFP
Innexin 8 rescue: ShakB2; VT50600.p65AD/UAS-ShakB; VT44845.Gal4DBD/UAS-myr::GFP

Immunohistochemistry: Standard confocal microscopy and immunocytochemistry methods were performed. In short, brains from 4-5 day old female flies were dissected in ice cold hemolymph-like buffer and fixed with 4% paraformaldehyde for 40 minutes, depending on age. The tissue was exposed to normalized donkey serum block for either 40 minutes at room temperature or overnight at 4C. After, the tissue was exposed to a primary antibody solution overnight at 4C. After being washed with 0.3% PBST, samples were exposed to a secondary antibody solution overnight at 4C. After a series of dehydration, tissue was mounted with DPX.

Primary antibodies used: Rabbit anti-Hunchback (1:400; Doe lab); Rat anti-HA (1:100; Sigma #11867423001; RRID AB_2687407); chicken anti-V5 (1:800; Fortis Life Sciences A190-118A; RRID AB_66741); mouse anti-Flag (1:1000; Sigma F1804; RRID AB_262044); Mouse anti-Lar (1:50; Developmental Studies Hybridoma Bank 9D82B3; RRID AB_528202); Mouse anti-Dlp (1:100; Developmental Studies Hybridoma Bank 13G8; RRID AB_528191); Guinea pig anti-Inx8 (1:1000; Desplan lab); rabbit anti-GABA (1:200; Sigma A2052; RRID AB_477652). Secondary antibodies were from Jackson ImmunoResearch (Donkey, 1:400).

bioRxiv preprint doi: https://doi.org/10.64898/2026.02.26.708359; this version posted March 2, 2026. The copyright holder for this preprint (which was not certified by peer review) is the author/funder, who has granted bioRxiv a license to display the preprint in perpetuity. It is made available under aCC-BY 4.0 International license.

Image acquisition and processing: Confocal image stacks were acquired on a Zeiss LSM900 Airyscan 2 microscope using a Plan-Apochromat 63x/1.4 Oil DIC M27 objective. Nyquist sampling, a pinhole of 1 Airy Unit, and optimal Z-stack step sizes were used. The imaging parameters were optimized to fill the detector's dynamic range while avoiding pixel saturation.

Super resolution Airyscan imaging was done for all Lar, Dlp, and Inx8 staining. For these images, the scan zoom was set to 1.3X and pixel scaling was set to 0.043 µm X 0.043 µm X 0.170 µm. All images were obtained with a frame averaging of 4 and a pixel dwell time of approximately 1.15 µsec. All images were processed either in Fiji (https:://imagej.new/fiji) or Imaris version 10.2 (https://imaris.oxinst). Methodology for specific analyses using these software packages are described below. Figures were made using Adobe Illustrator.

Quantification of cell body clusters: A cell body cluster was defined as 2 or more cell bodies that were touching. The number of cell body clusters were manually counted. The distance between cell body clusters was calculated by averaging the shortest distances between all the cell body clusters in a single animal.

Quantification of pixel intensity: All pixel intensity quantification was done manually using the "Measure" feature in Fiji. The freehand tool was used to outline the cell body. Measurements were set to "Area" and "Raw Integrated Density". A middle slice of the total cell body was measured. For each cell body, the "Raw Integrated Density" was divided by the "Area". The sum of these values is reported.

Quantification of Innexin 8 puncta: Imaris image analysis software was used to put a surface over the MDN cell body and the Inx8 staining. The "surface on surface" feature was used to isolate all the Inx8 surfaces on the MDN cell body surface. By selecting the Inx8 on MDN surfaces, Imaris reported the total volume and total number of Inx8 puncta.

Larval optogenetic behavior: Larval behavior methodology was described previously[3].

Adult optogenetic behavior: For adult optogenetic behavior experiments, all-trans retinal (ATR; Sigma-Aldrich Cat.# R2500) stock solution (100 mM) was prepared by dissolving 100 mg in 3.52 mL of 100% ethanol. Food vials were supplemented with 200 µL of 20 mM ATR and allowed to dry before setting crosses. Experimental and control flies were reared in the dark on ATR-supplemented food from eclosion until testing at 4–5 days old. Individual female flies were placed in a 1 mL serological pipette and allowed to acclimate for 2 minutes. MDN was optogenetically activated using a

bioRxiv preprint doi: https://doi.org/10.64898/2026.02.26.708359; this version posted March 2, 2026. The copyright holder for this preprint (which was not certified by peer review) is the author/funder, who has granted bioRxiv a license to display the preprint in perpetuity. It is made available under aCC-BY 4.0 International license.

627 nm red LED delivered from above for 5 seconds per trial, with a minimum 10-second inter-trial interval. Videos were recorded and locomotor behavior was scored blind to genotype. Forward and backward movement was determined relative to the fly's heading direction and averaged across trials. Flies that did not move during any trial were excluded. All behavioral experiments were performed at 22–25°C.

Full genotypes for these experiments; control genotype: UAS-CsChrimson.mVenus; VT50600.p65AD/+; VT44845.Gal4DBD/UAS-Luc RNAi TRiP.JF01355

Hunchback knockdown genotype: UAS-CsChrimson.mVenus; VT50600.p65AD/+; VT44845.Gal4DBD/ UAS-Hb RNAi TRiP.HMS01183

Lar knockdown genotype: UAS-CsChrimson.mVenus; VT50600.p65AD/ UAS-Lar RNAi TRiP.HMS02186; VT44845.Gal4DBD/+

Inx8 knockdown genotype: UAS-CsChrimson.mVenus; VT50600.p65AD/+; VT44845.Gal4DBD/ UAS-shakB RNAi TRiP.JF02604

Multicolor FlpOut and quantification of morphology: Genetics are explained in detail elsewhere[4]. Adult flies less than 24 hours old were heat shocked at 37°C for 8-12 minutes in a water bath. After the heat shock, they recovered at 18°C for an equal amount of time. The brain was dissected from 4-5 day old animals. Images of the brains were imported into the Imaris Image Analysis Software. The filament tool was used to trace the morphology of MCFO-labeled MDNs. Defining the primary neurite as the neurite extending from the cell body, the ipsilateral dendrite was the material ipsilateral to, but not including the primary neurite. The contralateral dendrite was the material contralateral to, but not including, the primary neurite. The first projection off the descending neurite was used as a landmark for the beginning of the axon region and the end of the contralateral dendrite region, similar to the larval MDN[3]. Only axonal material protruding off the primary neurite in the individual thoracic segments were reported. Total material was defined as the sum of all filaments in the region of interest. Number of bifurcations was defined as the number of times a filament split from one to two branches in the region of interest.

Full genotype for these experiments; control genotype: hs-FlpG5::Pest; 10X UAS(frt.Stop)myr::smGDP-V5-THS-10XUAS(frt.Stop)myr::GDP-Flag/VT50600.p65AD; VT44845.Gal4DBD/UAS-Luc RNAi TRiP.JF01355

Hunchback knockdown genotype: hs-FlpG5::Pest; 10X UAS(frt.Stop)myr::smGDP-V5-THS-10XUAS(frt.Stop)myr::GDP-Flag/VT50600.p65AD; VT44845.Gal4DBD/ UAS-Hb RNAi TRiP.HMS01183

Quantification of synapses: To quantify the total number of pre-synapses on MDN in the ventral nerve cord, we expressed a tagged non-functional version of the pre-synaptic protein bruchpilot (Brp) in MDN. Using Imaris image analysis software, the

bioRxiv preprint doi: https://doi.org/10.64898/2026.02.26.708359; this version posted March 2, 2026. The copyright holder for this preprint (which was not certified by peer review) is the author/funder, who has granted bioRxiv a license to display the preprint in perpetuity. It is made available under aCC-BY 4.0 International license.

spots tool was used to unbiasedly label the pre-synaptic puncta with a 3-dimensional spot.

Full genotypes for these experiments; control genotype: LexAop-myr::smGdp-V5, UAS-myr::smGdp-HA; VT50600.p65AD/UAS-brp-Short::mCherry; VT44845.Gal4DBD/ UAS-Luc RNAi TRiP.JF01355

Hunchback knockdown genotype: LexAop-myr::smGdp-V5, UAS-myr::smGdp-HA; VT50600.p65AD/UAS-brp-Short::mCherry; VT44845.Gal4DBD/ UAS-Hb RNAi TRiP.HMS01183

Genetically encoded voltage indicator experimental setup and analysis: Flies were reared in the dark prior to these experiments. At one day after emerging from the pupal case, adult flies were transferred to food supplemented with 5 mM all-trans retinol (ATR; Sigma-Aldrich Cat.# R2500).

We utilized an explant setup. At 4 and 5 days old, a brain was dissected and immediately placed in a puddle of hemolymph-like buffer (HLB) on a poly-lysine coated coverslip (Corning, Cat.# 354085). The coverslip was placed HLB puddle side-up on a slide. This setup works well for short periods of time (<5 minutes). HLB is made in-lab and is comprised of the following: 70mM NaCl, 5mM KCl, 1.5mM $CaCl_2$, 4mM $MgCl_2$, 115mM sucrose, 5mM HEPES, 10mM $NaHCO_3$, and 5mM trehalose.

The slide was then placed under the 3i 2-photon microscope with Coherent Chameleon Discovery IR laser and imaged using a water-dipping Zeiss Plan Apochromat DIC VIS IR 20X/1.0 objective in bi-directional resonant scanning mode. The excitation laser was tuned to 920 nm at a power setting of 24, and an emission filter of 525/50 was used. The detector gain was set to fill the dynamic range of the detector without saturating pixels. HLB was used in replacement of water, and more was added to the original puddle as needed. A region of interest of 396 by 28 pixels at a pixel scale of approximately 1.4 was drawn around the cell bodies. A spatial light modulator was used to stimulate a single cell body with red light, which was presented from 50 to 200 milliseconds. The total length of the recording was 20 seconds, imaged at 330 frames/second. All other parameters described previously were followed[5].

Suite2P was used to analyze the videos[6]. Raw fluorescence signals were extracted and normalized to the baseline fluorescence prior to the red-light stimulus. $\Delta F/F$ was calculated as the percent change from baseline, and traces and summary data were visualized accordingly. A Pearson correlation coefficient was calculated between the Stim and no-Stim groups for each animal to determine their likelihood of having similar voltage responses.

Full genotypes for these experiments; control genotype: UAS-Chrimson::mVenus; VT50600.p65AD/ UAS-ASAP5; VT44845.Gal4DBD/ UAS-Luc RNAi TRiP.JF01355

bioRxiv preprint doi: https://doi.org/10.64898/2026.02.26.708359; this version posted March 2, 2026. The copyright holder for this preprint (which was not certified by peer review) is the author/funder, who has granted bioRxiv a license to display the preprint in perpetuity. It is made available under aCC-BY 4.0 International license.

Hunchback knockdown genotype: UAS-Chrimson::mVenus; VT50600.p65AD/ UAS-ASAP5; VT44845.Gal4DBD/ UAS-Hb RNAi TRiP.HMS01183
Inx8 knockdown genotype: UAS-Chrimson::mVenus; VT50600.p65AD/ UAS-ASAP5; VT44845.Gal4DBD/ UAS-shakB RNAi TRiP.JF02604

Statistics: When applicable, values were normalized to the average of the control. All statistical analysis were performed with Prism 10 (GraphPad Software, San Diego, CA) or Venny (https://bioinfogp.cnb.csic.es/tools/venny/index.html). Numerical data in graphs show individual measurements (dots) and means (bars). The number of replicates (n) and definition of measurement reporter (i.e. animal, axon) for each data set is in the corresponding legend.

bioRxiv preprint doi: https://doi.org/10.64898/2026.02.26.708359; this version posted March 2, 2026. The copyright holder for this preprint (which was not certified by peer review) is the author/funder, who has granted bioRxiv a license to display the preprint in perpetuity. It is made available under aCC-BY 4.0 International license.

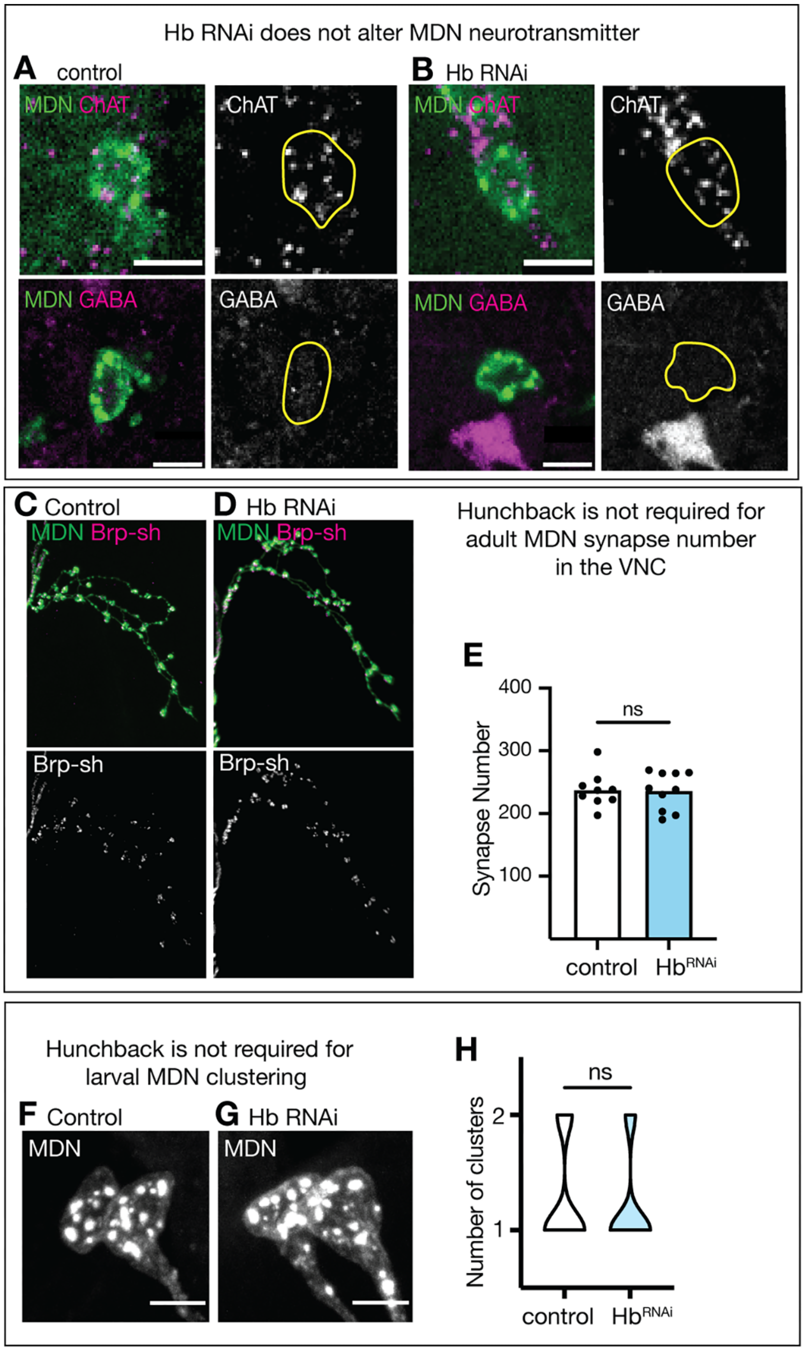

Figure S1. Hunchback does not regulate neurotransmitter identity or larval cell body clustering.

(**A, B**) ChAT (upper) and GABA (lower) expression. (**C, D**) MDN pre-synapses tagged with Brp-Short in the T1 segment of the VNC. (**E**) Number of MDN synapses in the VNC. Statistics: t-test, $p = 0.8287$. (**F, G**) Larval MDN cell body location. Scale bar, 5 µm. (**H**) Number of cell body clusters. Statistics: t-test, $p = 0.7744$.

bioRxiv preprint doi: https://doi.org/10.64898/2026.02.26.708359; this version posted March 2, 2026. The copyright holder for this preprint (which was not certified by peer review) is the author/funder, who has granted bioRxiv a license to display the preprint in perpetuity. It is made available under aCC-BY 4.0 International license.

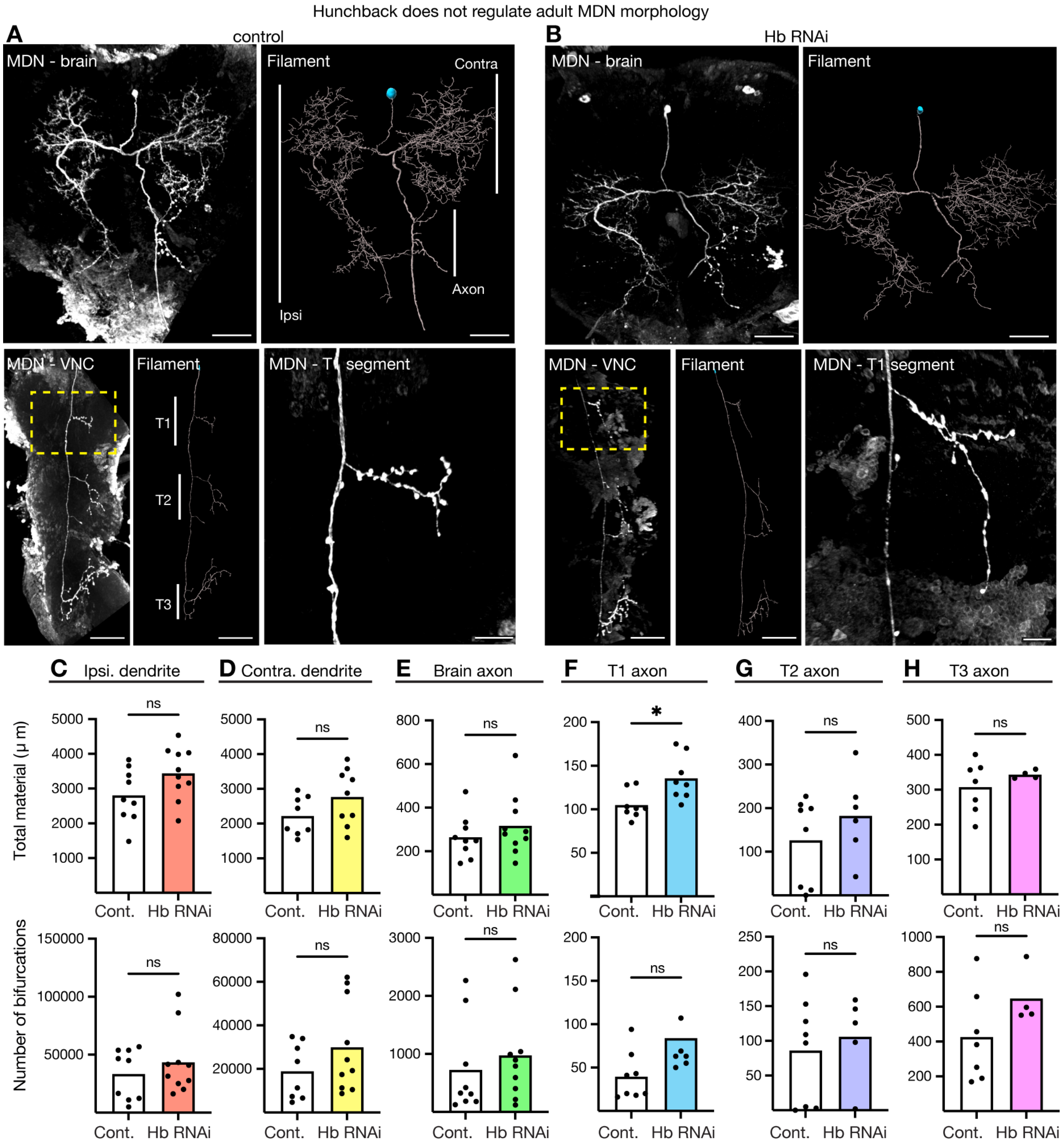

Figure S2. Hunchback does not regulate individual MDN morphology.
(**A, B**) *In vivo* and Imaris Filament morphology in MDN brain (scale bar, 30 μm), VNC (scale bar, 50 μm), and T1 segment (scale bar, 10 μm). (**C-H**) Total material (upper) and number of bifurcations (lower) across regions in the central nervous system. Statistical analyses were performed using unpaired two-sided t-test (*$p<0.05$).

bioRxiv preprint doi: https://doi.org/10.64898/2026.02.26.708359; this version posted March 2, 2026. The copyright holder for this preprint (which was not certified by peer review) is the author/funder, who has granted bioRxiv a license to display the preprint in perpetuity. It is made available under aCC-BY 4.0 International license.

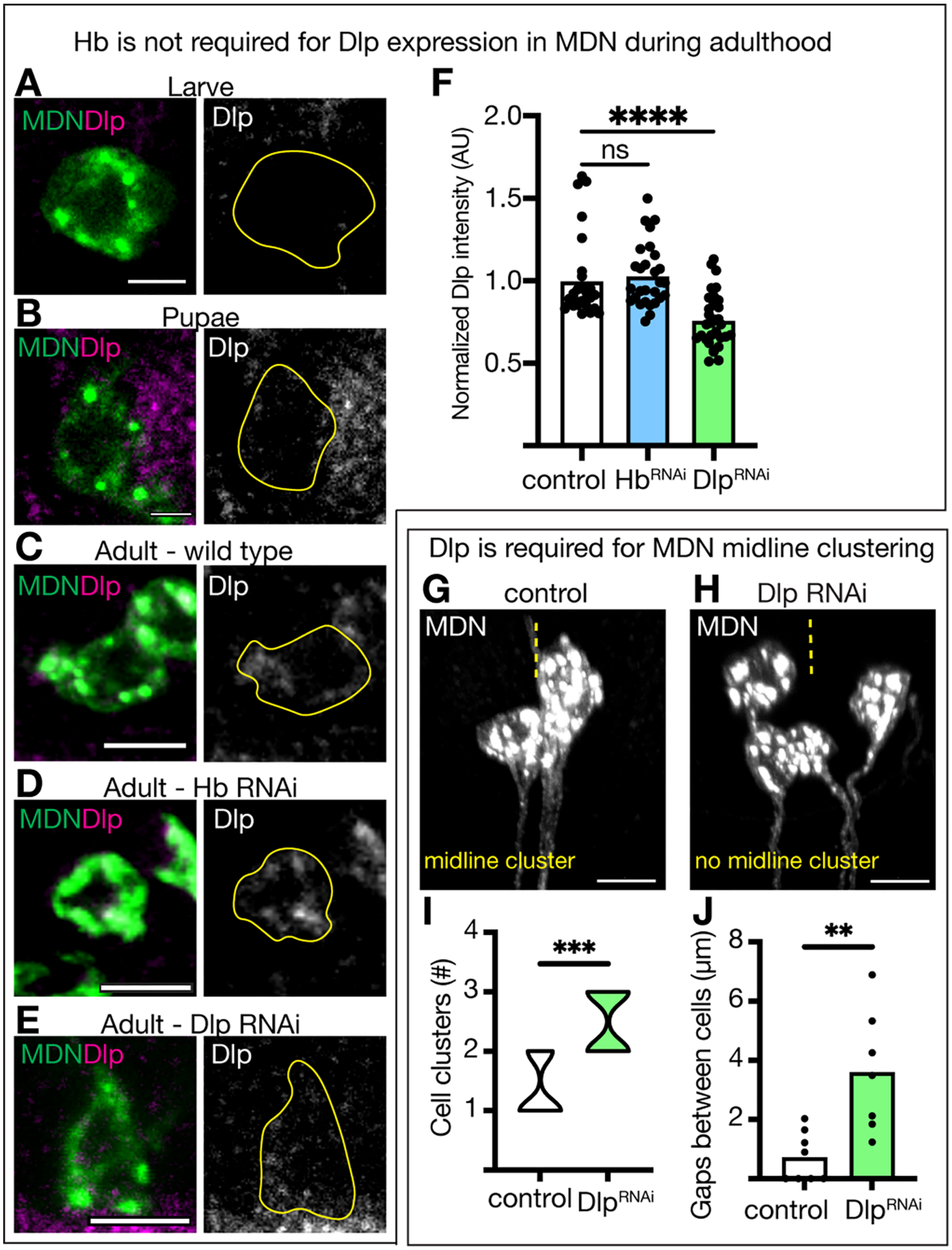

Figure S3. Dlp expression is required for MDN midline clustering.
(**A-C**) Dlp expression in MDN throughout life. A and C: scale bar, 3 µm. C: scale bar, 5 µm. (**D**) Dlp expression when Hb RNAi is expressed in MDN. Scale bar, 1 µm. (**E**) Dlp expression when Dlp RNAi is expressed in MDN. Scale bar, 1 µm. (**F**) Quantification of C-E. (**G, H**) Adult MDN cell body morphology. Scale bar, 7 µm. Yellow dash line represents the midline. (**I, J**) Quantification of G and H. Statistical analyses were performed using a two-way ANOVA with Bonferroni's multiple comparisons or an unpaired two-sided t-test, as appropriate (*p<0.05, **p<0.01, ***p<0.001, ****p<0.0001).

bioRxiv preprint doi: https://doi.org/10.64898/2026.02.26.708359; this version posted March 2, 2026. The copyright holder for this preprint (which was not certified by peer review) is the author/funder, who has granted bioRxiv a license to display the preprint in perpetuity. It is made available under aCC-BY 4.0 International license.

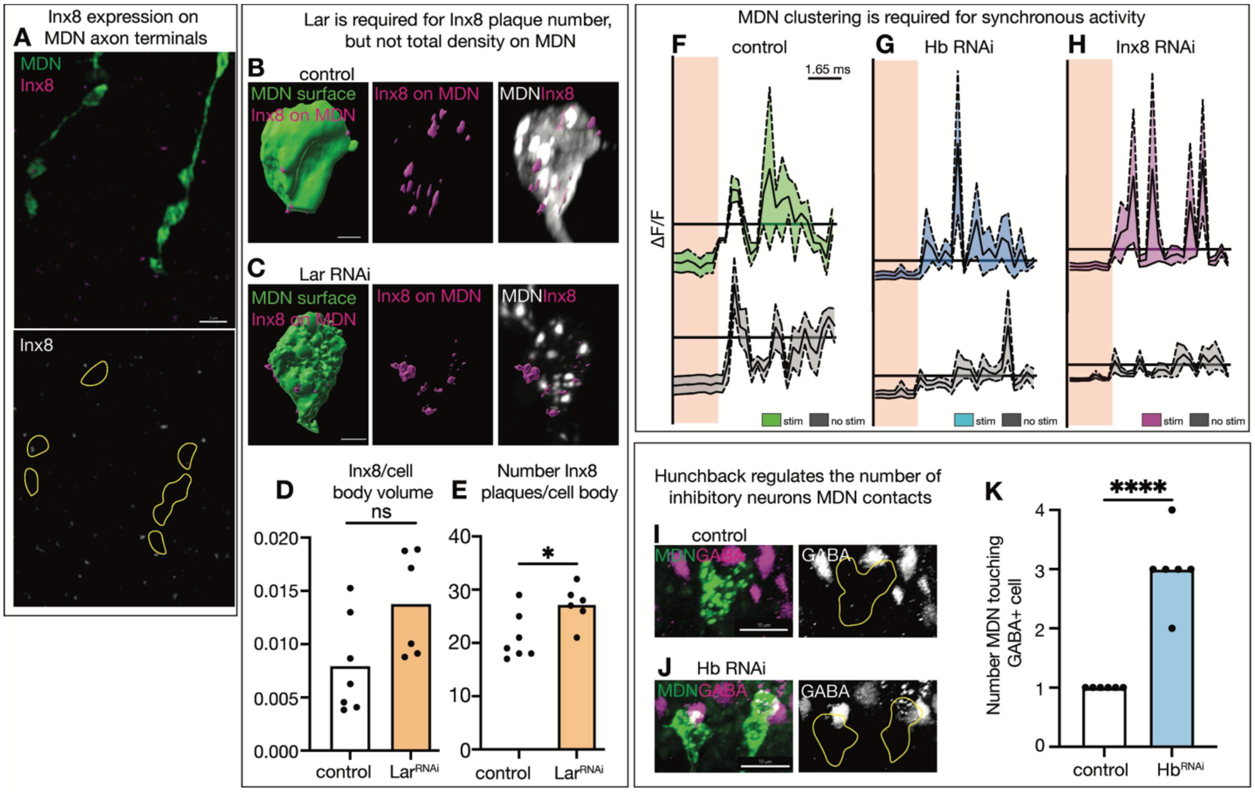

Figure S4. Inx8 expression, which is lacking on MDN axon terminals and smaller on MDN cell bodies when Lar is knocked down, between MDN cell bodies is required for synchronous activity.
(**A**) Inx8 expression on MDN axon terminals (yellow outline) in the brain. Scale bar, 2 µm. (**B, C**) Inx8 puncta expression on MDN cell bodies when Lar RNAi is expressed in MDN. Scale bar, 3 µm. (**D, E**) Quantification of B-C. (**F-H**) Voltage indicator traces between stimulated cell (colored) and adjacent cell (black) in total population of animals assayed when Hb and Inx8 RNAi is expressed in MDN. (**I, J**) GABA positive cells touching MDN cell bodies in when Hb RNAi is expressed in MDN. Scale bar, 10 µm. (**K**) Quantification of I-J. Statistical analyses were performed using unpaired two-sided t-test (*$p<0.05$).

bioRxiv preprint doi: https://doi.org/10.64898/2026.02.26.708359; this version posted March 2, 2026. The copyright holder for this preprint (which was not certified by peer review) is the author/funder, who has granted bioRxiv a license to display the preprint in perpetuity. It is made available under aCC-BY 4.0 International license.

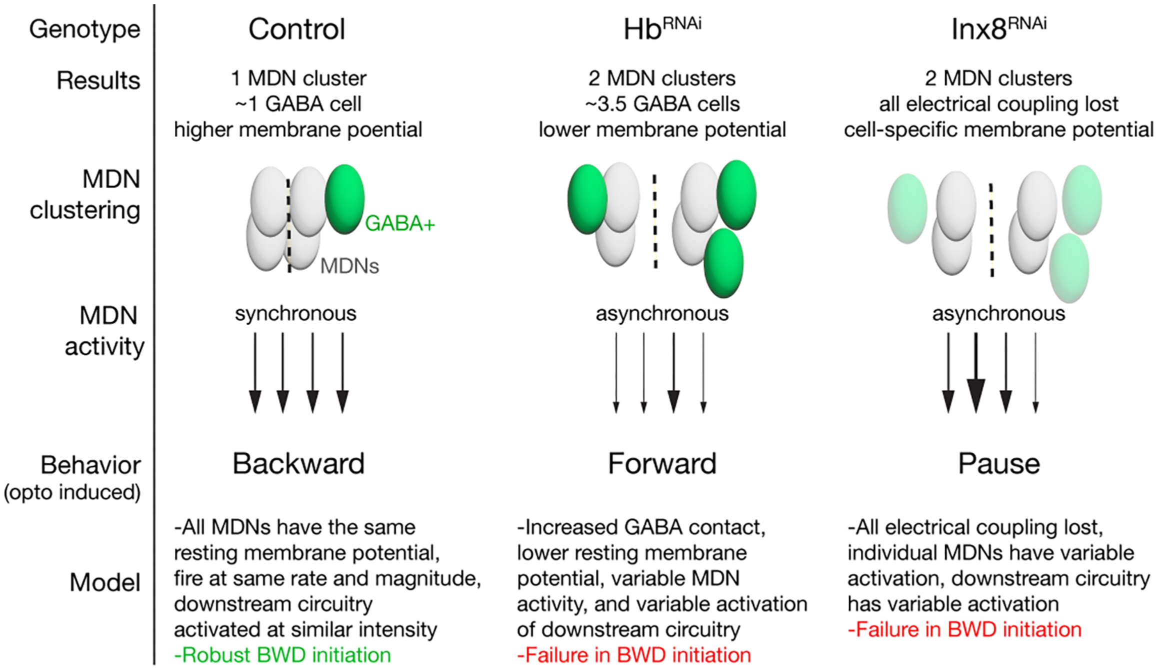

Figure S5. Summary of results.

bioRxiv preprint doi: https://doi.org/10.64898/2026.02.26.708359; this version posted March 2, 2026. The copyright holder for this preprint (which was not certified by peer review) is the author/funder, who has granted bioRxiv a license to display the preprint in perpetuity. It is made available under aCC-BY 4.0 International license.

References

1. Bidaye, S. S., Machacek, C., Wu, Y. & Dickson, B. J. Neuronal control of Drosophila walking direction. *Science* **344**, 97–101 (2014).

2. Feng, K. *et al.* Distributed control of motor circuits for backward walking in Drosophila. *Nat. Commun.* **11**, 6166 (2020).

3. Lee, K., Rico Carvajal, N., Graciani, J. & Doe, C. Q. Hunchback functions in the postmitotic larval MDN to restrict axon outgrowth, synapse formation, and backward locomotion. *Genes Dev.* genesdev;gad.353223.125v1 (2025) doi:10.1101/gad.353223.125.

4. Nern, A., Pfeiffer, B. D. & Rubin, G. M. Optimized tools for multicolor stochastic labeling reveal diverse stereotyped cell arrangements in the fly visual system. *Proc. Natl. Acad. Sci.* **112**, (2015).

5. Hao, Y. A. *et al.* A fast and responsive voltage indicator with enhanced sensitivity for unitary synaptic events. *Neuron* **112**, 3680-3696.e8 (2024).

6. Pachitariu, M. *et al.* Suite2p: beyond 10,000 neurons with standard two-photon microscopy. Preprint at https://doi.org/10.1101/061507 (2016).
